# Involving Medical Students in the Curriculum Development of Traditional, Complementary and Integrative Medicine: An Exploratory Qualitative Study

**DOI:** 10.1177/23821205251370544

**Published:** 2025-09-11

**Authors:** Eléonore Zurkinden, Julie Dubois, Pierre-Yves Rodondi, Benedikt M. Huber

**Affiliations:** 1 Institute of Family Medicine, Faculty of Science and Medicine, 98839 University of Fribourg, Fribourg, Switzerland; 2 Department of Community Health, Faculty of Science and Medicine, 27211 University of Fribourg, Fribourg, Switzerland; 3 Center for Integrative Pediatrics, Department of Pediatrics, Fribourg Cantonal Hospital, Fribourg, Switzerland

**Keywords:** curriculum development, student involvement, complementary medicine, integrative medicine, student-centered learning

## Abstract

**Background:**

Traditional, complementary and integrative medicine (TCIM) is acknowledged as integral parts of healthcare systems worldwide and thus increasingly integrated in medical education. Regarding undergraduate medical education, studies demonstrate the positive attitude of medical students toward TCIM and their general interest in it. However, their engagement in curriculum development has not yet been explored in this context.

**Method:**

We conducted an exploratory qualitative descriptive study using a focus group discussion with fourth-year medical students to explore perspectives, experiences, and expectations in relation to a novel curriculum on TCIM at Fribourg University in Switzerland. The aim was to identify elements that could be of general importance for curriculum development of TCIM in undergraduate medical education.

**Results:**

Main themes derived from the analysis were (a) the need for and usefulness of a curriculum about TCIM in undergraduate medical education, (b) satisfaction with the content and structure of the Fribourg curriculum, and (c) important competencies acquired or to be acquired during the curriculum. Most important, students agreed that TCIM is essential for all students and not just optional in medical school curricula. Students favored a longitudinal and transversal curriculum for TCIM, allowing cross-fertilization with other medical disciplines. Students emphasized the importance of a patient-centered and relationship-based approach to good patient care, which is integral to the definition of TCIM. Finally, students highlighted the diversity of attitudes, expectations, and perspectives as an inherent issue for academic teaching and learning as well as for patient care.

**Conclusion:**

Our study demonstrates the usefulness and importance of engaging students in undergraduate medical curricula on TCIM through participation in the process of continuous curriculum development. Medical students in our study consider TCIM to be an essential subject, which advocates for its stronger inclusion in medical training to prepare future doctors to provide patient-centered care in increasingly complex healthcare systems.

## Introduction

Integrative medicine (IM) describes a patient-centered, resource-oriented, and relationship-based approach that takes into account all appropriate methods from conventional and complementary medicine (CM) to promote health, healing, and development.^1–3^ CM encompasses a broad spectrum of disciplines and treatment methods including traditional healing systems that complement or expand those of conventional medicine.^4^ With regard to medical doctors’ education and practice, CM is usually taught and practiced in combination with conventional medicine in an integrated fashion. To stress this context, the term traditional, complementary and integrative medicine (TCIM) designates this integrated practice of CM within the framework of IM.^5^ TCIM is increasingly becoming an integral part of healthcare systems around the world, as evidenced by the widespread use of TCIM among the population^6–9^ and the growing interest in TCIM among healthcare professionals.^10–14^ There is an obvious need for education and information about TCIM for medical students, who will play a central role in the multiprofessional healthcare workforce in their future role as medical doctors. However, despite more than 25 years of CM/TCIM education research,^15–20^ debate continues on how to integrate teaching of TCIM into undergraduate curricula in medical schools.^20–26^

Several models have been developed during the last decades to promote TCIM teaching to medical students by using different formats, such as self-study, didactics, small-group interaction, interest-group activities, and wellness groups.^19,21,25,27^ Studies suggest that a multimodal curriculum significantly improves medical students’ familiarity with CM.^28^ Yet, as demonstrated by the review from Soliman and Bilszta,^24^ CM teaching in undergraduate medical education is widely inconsistent and not well aligned with clearly defined aims and objectives. The educational outcomes are very often unclear, especially with regard to clinical practice and/or patient outcomes.

In Switzerland, medical faculties are required to offer CM teaching during undergraduate medical education, which comprises the bachelor's and master's degree programs.^22^ This is based on the adoption of an article on CM in the Swiss Constitution by the Swiss population in May 2009.^29,30^ The constitutional article specifies that, within the scope of their powers, the Confederation and the Cantons shall ensure that consideration is given to CM (Article 118a of the Federal Constitution of the Swiss Confederation).^31,32^ Consequently, CM was incorporated into the Medical Professions Act and included in the learning objectives for students of human medicine, dentistry, chiropractic, veterinary medicine, and pharmacy.^33,34^ However, because implementation is left to the individual medical schools within the framework of university autonomy, the scope and nature of CM teaching at Swiss universities vary widely and there is no consensus on the way to teach it, the precise learning objectives, the knowledge and skills to be acquired, and the means to assess them.^22,35,36^

When a new master's degree program in human medicine was introduced at the University of Fribourg in 2019 (a bachelor's degree had been gradually introduced since 1896), it provided the opportunity to develop a completely novel undergraduate curriculum for TCIM. A first concept was built on an earlier course from the bachelor's program, on the personal expertise and experience of the teachers and leaders involved, and on evidence from the literature on TCIM education research.^25,37–39^ From the beginning, the aim was to create a longitudinal curriculum embedded in the entire bachelor's and master's programs. It spans from basic concepts, epidemiological and epistemological aspects, and introduction to major CM methods to the clinical application of CM approaches within the framework of IM. Implemented in 2021, the Fribourg Curriculum on Complementary and Integrative Medicine (FR-CCIM) currently consists of 10 hours of compulsory courses (lectures) and a 16-hour elective course on different CM methods (Table 1). In addition, the FR-CCIM supports interested students in finding internships and clinical rotations in medical practices and hospitals that offer TCIM and encourages early engagement in scientific research in the field of TCIM (master's and/or doctoral thesis).

**Table 1.** Overview of the Fribourg Curriculum on Complementary and Integrative Medicine (FR-CCIM) for the Undergraduate Medical Education at the University of Fribourg, Switzerland.

Academic year	Compulsory	Elective	Optional internship	Optional scientific work
**First**	**–**	**–**	**–**	**–**
**Second**	**–**	**–**	**–**	**–**
**Third**	4 h **lecture** on the principles of TCIM (1. Introduction to TCIM, epistemological aspects and classifications, 2. Overview of the main methods of CM, 3. Scientific research in TCIM, 4. Use and practice of TCIM)	**–**	**–**	**–**
**Fourth**	4 h **interactive seminar** on the practice of TCIM in various medical disciplines 2 h **course** on opportunities and risks of TCIM including ethical and health insurance aspects	16 h **elective course** on different CM methods including herbal medicine, traditional Chinese medicine, homeopathy, anthroposophic medicine, naturopathy, osteopathy, ayurvedic medicine	**–**	**Master's thesis** on TCIM
**Fifth**	**–**		**–**	
**Sixth**	**–**	**–**	**Internships/clinical rotations** in hospitals offering TCIM	**Doctoral thesis** on TCIM
			

*Note*: Bachelor's program corresponds to academic years 1 to 3, and Master's program to years 4 to 6.

In the context of the global curriculum development at the University of Fribourg, we decided to actively involve the students with the aim of further adapting and continuously improving this primary concept of the FR-CCIM. Studies have demonstrated that medical curricula can benefit from students’ contribution and that student involvement in medical curriculum development is a purposeful approach to empower students and foster student-centered learning contributing to personal and professional development.^40–44^ To that purpose, we conducted a qualitative study in order to explore the perspectives, experiences, and expectations of medical students in relation to the FR-CCIM, in terms of both content and form. This should help to align teaching more specifically with students’ needs and learning objectives. Furthermore, by enabling students to be part of the process of continuous curriculum development, this study sought to highlight the usefulness and importance of student engagement for curriculum design in the field of TCIM.

## Methods

### Study design

An exploratory qualitative descriptive study^45^ that used a focus group (FG) was conducted with fourth-year medical students at the University of Fribourg in Switzerland. FG discussion was chosen because it allows stimulation of conversation and interaction among participants.^46^ This method is used to obtain the knowledge, perspectives, and attitude of people about issues, and it is an opportunity to understand participants’ views and opinion toward a particular subject.

### Participants and recruitment

All fourth-year medical students of the academic year 2022 to 2023 (*n* = 36) were eligible to participate in the study regardless of whether they participated systematically in the FR-CCIM courses or not. Fourth-year medical students were chosen because they had just completed the compulsory courses of the FR-CCIM (Table 1). The study was presented to the students at the end of the last compulsory course in the spring semester 2023. In addition, all students subsequently received an email presenting the study and its objectives and inviting them to participate. To increase the recruitment rate, two follow-up emails were sent to students two weeks apart after the summer vacations. Students were informed that participation was voluntary and that the decision to participate or not would have no impact on the further course of their studies. Students interested in participating in the study then contacted the research team by using the given contact information. A date suitable for the majority of interested students was then set for the FG.

The Cantonal Commission for the Ethics of Human Research (CER-VD) waived the need for formal ethics approval for this study because no health-related personal data were collected (Reference Req-2023-00215). However, all procedures performed in this study were in accordance with the Swiss Federal Act on Research involving Human Beings and with the 1964 Helsinki Declaration and its later amendments or comparable ethical standards.^47,48^

### Data collection

An interview guide was developed by the study team and used during the FG (Supplementary File S1). Questions revolved around (a) students’ perceived interest in learning about TCIM, (b) whether the content of the FR-CCIM allowed them to reach the objectives set for the course, (c) students’ satisfaction regarding the content and format of the FR-CCIM, and (d) the influence of the FR-CCIM on their perception of TCIM.

The FG took place in September 2023 at the beginning of the fifth academic year in a classroom at the University of Fribourg during lunch time. The FG was moderated by JD (a senior qualitative researcher in primary care, MA). EZ (a primary care physician and teacher in the FR-CCIM, MD) was present to help with the organization, take note of the speaking turns, and manage the recorder. She was trained by JD in this task. JD and EZ presented themselves to the students, presented the objectives of the study, and explained the procedure of the FG. Oral informed consent to participate was collected from the students before starting the FG. The FG was audio-recorded and then transcribed verbatim.

### Data analysis

A thematic analysis of the transcripts was performed with the assistance of the qualitative data analysis software MAXQDA (v.2018.2).^49^ As a first step, JD read all the transcripts to familiarize herself with the data and establish a first set of codes (codebook). She then coded all the transcripts, and as the analysis went on, the codebook was adapted iteratively as new codes were identified in the data. Memos were written by JD to describe each code and ensure consistency in coding. The codes were then compared and similar codes merged and classified into larger themes and subthemes. The coding was mainly deductive, as the main themes were derived from the interview guide. Meetings were organized with all co-authors to discuss coding and interpretation of the data and to reach an agreement on the final structure of the coding. Apart from EZ and JD, the other co-authors were a primary care physician and professor in primary care (PYR, MD) and a pediatrician and co-director of the Center for Integrative Pediatrics at the Fribourg Cantonal Hospital (BH, MD). Both PYR and BH taught in the FR-CCIM.

Our data are based on quotations from participants. JD translated the quotations that are used in the manuscript from French to English. A native English speaker then read it an additional time to ensure that the idiomatic meaning of phrases of the FG participants was preserved. We used the COnsolidated criteria for REporting Qualitative research (COREQ) checklist to report our study (Supplementary File S2).^50^

## Results

Eight students (8/36, 22%) participated in a single FG discussion (duration: 83 minutes). Only two students (25%) had also attended the elective course on CM methods so far, which may indicate particular interest a priori in TCIM, whereas some students considered themselves to be or to have been rather skeptical about CM.

Three main themes were derived from the analysis: (a) need for and usefulness of a curriculum about CIM in undergraduate medical education, (b) satisfaction with the content and structure of the FR-CCIM, and (c) important competencies acquired or to be acquired during the curriculum.

### Need for and usefulness of a curriculum about CIM

#### Getting an overview

Throughout the entire FG discussion, all students emphasized the importance of having an overview of what CIM consists of, regardless of personal opinions on the subject. Most considered it a professional duty for any (future) physician to be informed about fundamental principles and formal aspects of CM.

“I think there are always those who are completely against it or completely for it, and we could do anything and they wouldn’t change their minds. But in the end, the reality is that it [use of CM] is something that's happening more and more often, and I think that if you want to be a good doctor in the years to come, you have to know something about it. Whether you believe in it or not, in the end.” (Student 7)

#### Being open to the subject

Similarly, students expressed that, given the widespread use of CM in the population, it is important to be open to the subject, both to foster the therapeutic relationship with their patients and to be aware of the treatments used by them.

“And also the fact of being accessible to patients. If patients know that we’re closed, definitively, to CM, I don’t think it will necessarily encourage them to come to the medical practice. Or they may turn to CM instead, perhaps forgetting certain important things.” (Student 3)

#### Having basic knowledge about different types of CM

They underlined that they should acquire a wide basis of knowledge rather than focusing on a particular CM. Students acknowledged that it was not possible to study each CM method, but felt that they should at least know about the principal types of CM or the most used by the population. They considered that further knowledge about CM could be acquired through practice experience or additional postgraduate training in CM.

“I think this also comes with experience, because you can't learn this or that situation in the classroom. But if you've already got a basic knowledge of what's out there, and maybe also a little knowledge of the diseases or people who tend towards this or that, that's not bad. But I don't think that at the moment I'd be able to recommend anything to a person that would be appropriate.” (Student 4)

#### Being provided with an additional toolbox

Students also pointed out that having teachings about CM might provide them with an additional toolbox for their future practice, notably in cases where conventional medicine reaches its limits or when confronted with patients with chronic conditions. In this context, too, they emphasized the importance of the therapeutic relationship.

“I think we can also add that if we ever have a patient who doesn't agree with our ideas, we might have an option to propose to them that we both agree with, and that way we can keep the relationship going.” (Student 4)

#### Medical practice versus hospital setting

Finally, students discussed the context for the practice of TCIM and most felt that teachings on TCIM were particularly useful for working in a medical practice, especially family medicine or pediatrics, compared with the hospital setting.

“A hospital is still a bigger setting, it's stricter with all that and in a practice there are more possibilities. You're also more alone, you have less supervision and so you have to find different ways of helping your patients. Because in hospital there's more of a clear-cut problem: you come in, you undergo surgery and you leave. At the practice, there are a lot of people who come in with pain and we can't do anything for them, so we still have to find a solution to help them.” (Student 1)

### Satisfaction with the content and structure of the curriculum (FR-CCIM)

Overall, students were satisfied with the content and structure of the curriculum developed so far. However, they also pointed out areas for improvement and voiced some critiques.

#### Adequate balance

Students considered that the amount of time dedicated to the FR-CCIM was sufficient and adequately balanced with regard to other disciplines and to the overall learning objectives for medical students. Some students felt that people interested in learning more about CM could attend the elective courses proposed by the curriculum or pursue additional postgraduate training in CM.

“I think more courses on CM would be… Especially in comparison… I mean, knowing all complementary medicines methods is interesting, but it's true that we already know almost nothing about the other professions in the hospital. So investing a lot of time in CM, when we don't know our colleagues’ jobs, is perhaps a bit… It might be a bit excessive. But I think it was a good balance.” (Student 3)

#### Structure of the FR-CCIM

Most students agreed on the timing of the FR-CCIM not starting before the third year of the bachelor program. They also appreciated that the teaching mostly took place before clinical rotations began and thus before being confronted with patients who potentially use CM. In addition, students underlined the benefits of having a transversal curriculum about TCIM, that is, that FR-CCIM courses were also integrated within thematic modules, such as chronic pain and oncology. This was judged as allowing them to look at the same subject from different perspectives and as reaching a wider audience of students.

“And I think it's interesting the way it's been integrated. We had, I don't know, a cardio block where we really had the cardiologists, then the point of view of family medicine, the point of view of integrative medicine. In fact, it wasn't just an integrative medicine block, where that's all we talk about. It was really integrated into what we were seeing and how it was being used. We were also able to compare different points of view quite quickly. I found that interesting. And I found that it applied to a certain extent to all the branches. So that's good.” (Student 8)

One even noted cross-fertilization with other courses outside of the FR-CCIM.

“Yes, even sometimes in courses that weren't about integrative or complementary medicine, we'd say ‘yeah, we can do that, too’, sometimes. Just to launch the idea a little. That was good, too.” (Student 1)

Finally, a few students explained how the FR-CCIM opened their minds regarding CM.

“I think it opened me up completely to CM, because it's something I didn't know, well I knew it by name but not much more than that. And then, maybe I'm too factual. But to see what it was in concrete terms, to see how it was applied, to see by whom it was applied, that clearly opened me up to it, and I find it really interesting. I think it's a really good thing to have seen that.” (Student 8)

#### Areas for improvement

Nevertheless, students highlighted some areas of improvement, both in terms of format and content of the courses. In terms of format, students proposed developing CM-specific clinical scenarios, or simulated patient scenarios, in order to be better prepared to care for patients who use CM, notably in cases of patients reluctant to use conventional medicine. One student also suggested that, as CM remains a controversial subject in medicine, debates could be organized to confront the differing views surrounding specific CM methods. Regarding content, some students advocated for more teachings around the financial (reimbursement policies, costs to the healthcare system, etc.) and cultural aspects of CM (eg about the types of medicine used by migrant populations in their home countries).

#### Main critiques

One main criticism toward the curriculum was the perceived lack of objectivity in certain courses or by certain teachers. However, opinions were divided on the matter. On the one hand, some students felt that a few courses were putting forward CMs without backing them up with sufficient evidence of their effectiveness.

“It's a subject that I find quite sensitive. Either we're inclined to listen, or we're more suspicious and we hold back. And it's true that I myself tend to hold back. But that's because we're trained to be evidence-based and that's it. And I find that it's a subject that is also, in some ways, quite political and… Yeah, that's right, you really get into the subjectivity of your personal assessment of a treatment. For me that's difficult to manage. You're no longer dealing with facts, you're dealing with ‘I think it works, I don't think it works’. And yeah, that's what bothers me a bit about CM. And that's why (…) it bothered me a bit that it was presented in a more subjective way.” (Student 3)

On the other hand, while acknowledging that evidence was sometimes missing for CM, other students did not necessarily see it as a negative point and were more interested in being informed about CM treatments than in being provided with data based on evidence exclusively. As one student summarized:

“A patient who is ill, who has a serious illness such as cancer, and who is in pain, I don't think he gives a damn about knowing that there is such and such a study which has proved that… In short, as long as he finds or we find something that can help him and as long as it remains within acceptable limits, I think that's what medicine is all about. (…) I think that as long as it works for the patient and it's not deviant or… It doesn't matter what you call it, it's better to go for it. So all that to say that studies and evidence are important (…) but that's not all either.” (Student 7)

Nonetheless, participants of the FG recognized the pluralism of perspectives and its importance for academic teaching and learning, as well as for treating patients. They acknowledged that students’ attitudes, expectations, and needs differ regarding didactic content and methods.

“But I do think that what's a bit difficult in a curriculum is finding the right balance between ‘we have to assert and prove to certain students who are very, very, very doubtful, that it can work’ and others who have less need for formal proof, that everything we're told about integrative medicine has to be proven by this or that study. And in fact, I think it's this balance that's really hard to find, because I don't think all students have the same expectations in relation to these courses. There are some who assume that – I'm exaggerating a little – but who assume that it doesn't really work anyway. The aim is to show them that yes, it can still be effective, and not to assume that no, it's not too effective. And then there are other students who might have different needs and interests, because they don't start from that principle.” (Student 6)

In the same sense, they discussed differences in patients’ values and needs that should to be taken into account when developing a treatment plan.

“And then, there are also clearly patients who, I think, then turn to people who may or may not think like them. There are patients who are hyper factual – well, in the few clinical rotations we’ve had, there are really patients who need to see, they’re very factual ‘why, why?’ and then there are others, on the contrary, who will really let themselves go, if you can put it like that. They’re more blindly trusting, and I think it’s the same for us. And there's no right or wrong. And I think it's important to work on both, to have everyone's different points of view, too.” (Student 8)

### Important skills and competencies acquired or to be acquired

#### History taking

The most cited competence acquired during the FR-CCIM was history taking. Students underlined the importance of asking patients systematically about their CM use, but some recognized that they would more readily do that in specific situations.

“But it is true that, as we were saying, alternative medicine targets the chronic rather than the acute. And I don’t think there are many people who break a leg and then think about doing something alternative, as a first-line treatment in any case. So, you can ask the question, but I think it has to be adapted to the context, as always.” (Student 7)

#### Identifying red flags and interactions

Another competence was the capacity to identify red flags in the context of CM use. Indeed, although students felt they did not learn a lot about the risks and benefits of CM, they did learn to recognize a series of situations that should raise concern, such as CM providers advising the patient to stop their conventional medication or not setting any objectives for the CM therapy.

“At the medical practice, we’ve had several occasions where patients have asked ‘well, in this situation, what CM can I use?’ or have suggested something they’d really like to do. And each time, I think that the important thing was to give them the red flags, to tell them what they should look out for in such and such a case.” (Student 3)

Finally, students felt that they had learnt to be aware of potential drug interactions when CM and conventional medicine were used concomitantly by their patients.

#### Important skills and competencies to be acquired

Participants of the FG had difficulty identifying other skills or competencies that they should acquire during the curriculum. They considered that the most important competence to be acquired through this curriculum was communication and the ability to inform patients, notably in terms of risks and benefits of CM therapies.

“I think that the central point remains the patient. You have to go where the patient wants to go. If the patient doesn’t want drugs, they want CM, and it’s our job—or at least I feel this way—to find out what’s best for the patient in terms of the treatment they want. And I think we’re talking more and more about CM and there’s more and more CM that’s reimbursed by complementary health insurance and all that, so I think it’s our duty to inform ourselves about that.” (Student 8)

In addition, students felt it was important to have a network to which they could refer their patients.

“And perhaps also to have a network, in other words people to whom we can refer cases. And then not telling patients ‘Yes, you can do that, but I don’t know anything about it and I don’t know if I can trust this professional or not.’” (Student 4)

## Discussion

The FR-CCIM aims to implement teaching on TCIM into the undergraduate medical education program at the University of Fribourg in Switzerland and thus to contribute to preparing medical students for their future tasks as physicians. We were able to demonstrate that students welcomed the content and structure of the FR-CCIM, which begins with a broad introduction and the basic principles, and then presents an overview of the different types of CM and their clinical application in different fields of medicine. Challenges in identifying competencies to be acquired could reflect the fact that the FR-CCIM did not yet include formal skills training.

The concept of student-centered learning offers an established and promising approach to curriculum development.^51,52^ By exploring students’ perspectives, experiences, and expectations, our study provides perspectives for future adaptation and improvement of the FR-CCIM. Moreover, it reveals specific points and key elements that are of general importance for curriculum development of TCIM in undergraduate medical education.

Students agreed that TCIM is an essential and indispensable topic for all medical students and not just an optional subject in medical school curricula. This shows recognition of the widespread use and demand for TCIM and the growing body of evidence from research on CM therapies, which requires medical doctors to have at least basic knowledge of TCIM to guarantee optimal and safe patient care. Our findings support the idea that at least the principles and concepts of TCIM should be delivered in compulsory courses to all medical students. This result is in line with results from previous studies^12,53–55^ and with views from academic leaders and experts.^20,27,36,56–58^ The literature also advises complementing these compulsory courses with electives on (major) CM methods and their clinical application,^21,22,24^ as is the case in the FR-CCIM.

Students also favored a longitudinal and transversal structure of a TCIM curriculum with courses embedded in the entire medical school program. This design allows coordination of content both within and outside the TCIM curriculum and may foster cross-fertilization with other medical disciplines. It confirms other didactic programs already established for the teaching of TCIM.^21,53^

An exciting new perspective for the further development of TCIM teaching emerged from the discussion of participants on the idea of integrating TCIM topics into more clinical courses of the undergraduate medical curriculum to stimulate clinical reasoning on a broad level that considers different perspectives. This suggestion resonates with that from experts, who have underlined the importance of combining conventional and CM procedures to foster IM treatment concepts in teaching programs.^56^ Our study underlines that such an approach could truly be the next level of integrating TCIM into medical education by recognizing TCIM as an integral part of today's healthcare systems. A promising didactic format, as also suggested by students in our study, could be clinical case scenarios presented and discussed by experts from different perspectives.^59,60^

Students further emphasized the importance of a patient-centered and relationship-based approach to good patient care. These elements are integral to the definition of IM,^1–3^ and TCIM curricula are thus excellently suited to teach these values, which are important for medicine as a whole.

Clinical expertise plays a crucial role in curriculum planning with regard to the selection of suitable teachers. In particular in the context of undergraduate medical education, one critique is that CM teaching is often provided by CM practitioners who lack a professional scientific background.^58^ Although the use of community-based CM practitioners is possible, both Soliman and Bilszta^24^ and Quartey et al^39^ describe the advantages of involving faculty members in teaching, especially if they have dual training in conventional medicine and CM. This could influence the perceived objectivity of the teaching of CM, which was a major point of criticism for the students in our study. The FR-CCIM tends to achieve this. However, still too few faculty members hold such dual training.

Evidence-based medicine (EBM) is often stressed in the context of TCIM education.^12,21,24^ Students in our study acknowledged its importance; however, they underlined that EBM should not be limited to external evidence from scientific research but should also consider the other two pillars of EBM, namely individual clinical expertise and patient values and preferences,^61–63^ to achieve optimal individualized patient care. Evidence from basic sciences and clinical research, as well as its critical appraisal, are necessarily required in courses on TCIM. In addition, TCIM curricula could offer students the opportunity to get involved in research in the field of TCIM at an early stage. The FR-CCIM, for example, actively promotes this in the form of master's and doctoral theses (Table 1).

As also shown in previous research,^12,55^ students in our study highlighted competencies such as history taking and communication in the context of TCIM for effective and safe patient care. This is an important point, as previous studies revealed that students felt uncomfortable talking about CM with patients and that the level of TCIM education in medical schools did not enable students to reach their goals.^53,64,65^ However, as some students considered that CM history taking was more important with chronic conditions, the curriculum should remind them that CM may be used for both chronic and acute conditions. Beyond teaching students to identify red flags related to patient CM use, TCIM courses could include communication skills training with real or simulated patients, as suggested by students in our study. In this context, setting clear treatment objectives for CM therapies and discussing the risks and benefits of CM therapies would be important learning outcomes. Furthermore, these training sessions would offer the opportunity to simulate situations that should raise concern, such as different and conflicting treatment recommendations or loyalty conflicts on the part of patients between different practitioners.

Finally, students emphasized the diversity of attitudes, expectations, and perspectives as an inherent issue for academic teaching and learning as well as for patient care. Considering this issue in medical education and curriculum design could prepare medical students to be aware of different approaches, treatments, and institutions that people use to maintain health or treat illness, which is sometimes described as medical pluralism.^66^ Notably, TCIM strives to overcome the often seemingly arbitrary pluralism in favor of a rational plurality of complementary directions and approaches in thinking and practice within medicine as a meaningful whole, one that is oriented toward patient needs and not toward disciplinary vanities.^67^

Against the background that general guidelines for comprehensive TCIM training in medical education are still often lacking, as it is shown for example by the learning objectives for medical students and faculties in Switzerland,^34^ the findings of our study could make a valuable contribution to better defining key elements for the form, content and objectives of TCIM training and to achieving a more consistent delivery of relevant TCIM training.

This study has some limitations. First, the presentation of the study took place during the last course on CM just before the summer break and the emails were subsequently sent out during the same vacation period. It is therefore possible that some students could not be reached during the summer break, which could have had an impact on recruitment and therefore on the number of participants (sample size). Despite email reminders, it was not possible to recruit enough students to conduct two focus groups, as originally planned. Second, given the recruitment strategy, it is possible that a selection bias toward students in favor of CM was present. However, because several students present described themselves as being skeptical about CM, it seems that the group was heterogeneous in terms of their basic attitude toward the topic. Third, conducting the FG with fourth-year students did not allow evaluation of the entire FR-CCIM, including electives, internships, and optional research activities (master's or doctoral thesis) in the fifth and sixth academic years. Finally, the authors of this study were all involved in FR-CCIM teaching, except for JD. Thus, it cannot be ruled out that this may have impeded students from truly voicing their opinions during the FG. To avoid any such bias, we reiterated at the start of the FG that the aim of the interview was to obtain candid accounts of teaching, whether positive or not.

## Conclusion

Involving medical students in curriculum development offers exciting opportunities to promote student-centered learning by taking into account student needs and expectations. Our study demonstrates the benefit of student engagement in undergraduate medical curricula on TCIM. Medical students in our study consider TCIM to be an essential subject, which advocates for its stronger inclusion in medical training to prepare future doctors to provide patient-centered care in increasingly complex healthcare systems. The proposal for an even more integrative teaching of CM in all clinical courses of the undergraduate medical curriculum and the consideration of the divergence of students in terms of expectations, objectives and learning approaches opens up additional perspectives for curriculum design. The FR-CCIM is an example of concrete implementation in the form of a longitudinal and transversal curriculum, with many possibilities for further improvement. Follow-up studies are needed to validate the current findings and to evaluate adjustments of the curriculum. Giving students an active part in the process of continuous curriculum development is in line with the overall concept of the FR-CCIM emphasizing and encouraging students’ own initiative for self-determined learning and personal development. Further research on TCIM curriculum development is warranted and should also include perspectives of faculty members and experts in the field of TCIM.

## Supplemental Material

sj-docx-1-mde-10.1177_23821205251370544 - Supplemental material for Involving Medical Students in the Curriculum Development of Traditional, Complementary and Integrative Medicine: An Exploratory Qualitative Study Supplemental material, sj-docx-1-mde-10.1177_23821205251370544 for Involving Medical Students in the Curriculum Development of Traditional, Complementary and Integrative Medicine: An Exploratory Qualitative Study by Eléonore Zurkinden, Julie Dubois, Pierre-Yves Rodondi and Benedikt M. Huber in Journal of Medical Education and Curricular Development

sj-docx-2-mde-10.1177_23821205251370544 - Supplemental material for Involving Medical Students in the Curriculum Development of Traditional, Complementary and Integrative Medicine: An Exploratory Qualitative Study Supplemental material, sj-docx-2-mde-10.1177_23821205251370544 for Involving Medical Students in the Curriculum Development of Traditional, Complementary and Integrative Medicine: An Exploratory Qualitative Study by Eléonore Zurkinden, Julie Dubois, Pierre-Yves Rodondi and Benedikt M. Huber in Journal of Medical Education and Curricular Development
