# Supplementary material for: Involving Medical Students in the Curriculum Development of Traditional, Complementary and Integrative Medicine: An Exploratory Qualitative Study
Source: J Med Educ Curric Dev. 2025 Sep 11;12:23821205251370544. doi: 10.1177/23821205251370544 (PMC12432310; doi:10.1177/23821205251370544)
Supplement: sj-docx-1-mde-10.1177_23821205251370544 - Supplemental material for Involving Medical Students in the Curriculum Development of Traditional, Complementary and Integrative Medicine: An Exploratory Qualitative Study [file sj-docx-1-mde-10.1177_23821205251370544.docx]

# Supplemental material

**Supplementary file 1.** Interview guide used during the focus group discussion (original version in French, translated into English for publication).

| **Topic** | **Questions** | **Follow-up questions** |
| --- | --- | --- |
| **Interest in the education**  **(benefits of education)** | In your opinion, what is the point (interest) of receiving education on complementary and integrative medicine?  In your opinion, what knowledge and skills should a medical student have concerning complementary and integrative medicine at the end of their studies? Why?  To what extent do you think that the courses of the curriculum enabled you to acquire this knowledge and these skills? |  |
| **Learning objectives** | To what extent do you think that the education you received in the FR-CCIM enabled you to achieve the objectives set out in the PROFILES [catalogue of learning objectives]?  To what extent do you think that the education you received in the FR-CCIM enabled you to achieve the objectives set out in the FR-CCIM? | Are there any items missing?  Are there any superfluous items? |
| **Program and teaching format** | Here is the current teaching program of the University of Fribourg (see Table 1)  What do you think of this program?  In general, how do you think TCIM should be taught: separately from other courses or, in contrast, by integrating them into the various general courses (e.g. case-based seminars)?  To what extent do you feel that the teaching provided enabled the specific objectives of each course to be achieved? | What do you think of the way the teachings are spread across the academic years?  What do you think of the number of hours dedicated to this topic (compared to other topics)?  Are there any items missing?  Are there any superfluous items?  Any suggestions for improving/ developing the curriculum for TCIM? |
| **Influence of the teachings** | To what extent do you think that the education you received during your studies will influence the way you deal with the issue of complementary medicine with patients? In what way?  To what extent do you think that the education you received has changed your perception of your own health?  To what extent do you think that the education you received has changed your perception of medicine in general?  Do you have any other comments? | What benefits/drawbacks do you see in the use of complementary and integrative medicine for the healthcare system?  What benefits/drawbacks do you see in the use of complementary and integrative medicine for patients? |
